# World Brain Day 2021 ‘Stopping MS’: an interview with Tissa Wijeratne and Joanna Laurson-Doube

**DOI:** 10.1038/s42003-021-02403-8

**Published:** 2021-07-22

## Abstract

The theme for World Brain Day (WBD) this year is ‘Stopping MS’. Despite the amazing progress that science and medicine have made in the development of therapies for multiple sclerosis (MS), access to such therapies is still a major challenge in many parts of the world. We spoke to Professor Tissa Wijeratne, one of the founders of WBD, who has steered many initiatives that aim to improve brain health globally and Dr Joanna Laurson-Doube about the actions needed to improve MS treatment worldwide.

**Figure:**
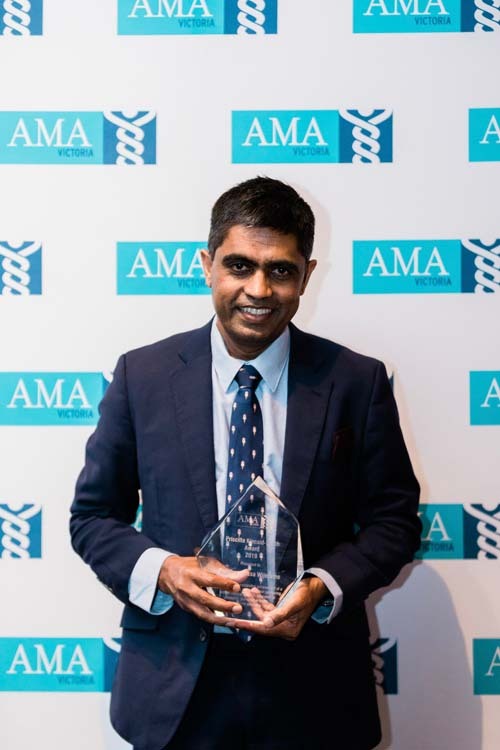
Tissa Wijeratne

**Figure:**
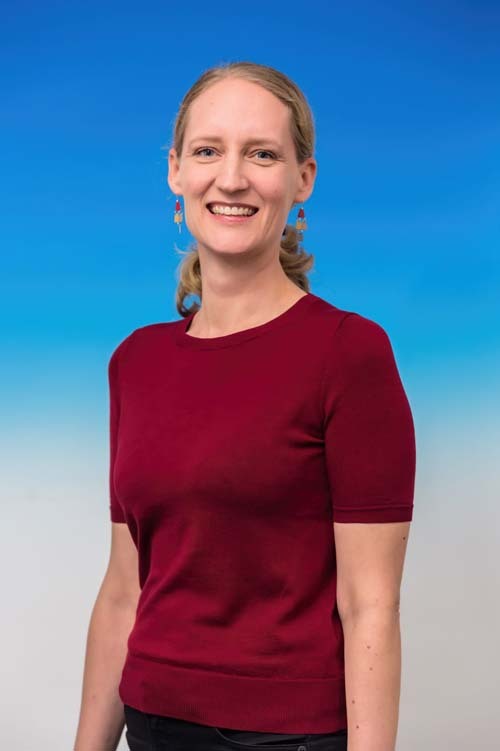
Joanna Laurson-Doube

Professor Tissa Wijeratne is one of the founders of WBD. His research interests in brain health are wide. One interest is reperfusion therapies in Stroke. He is part of the Melbourne stroke research group and has published several landmark papers in NEJM and Lancet that changed how stroke is managed. He also leads a research group in Melbourne, which was the first group in the world to demonstrate shared pathobiologies between stroke and COVID-19 associated brain involvement and as such, they coined the term Post Covid-19 Neurological Syndrome (PCNS). Tissa’s work has also included the description of a novel prognostic index named Serial Systemic Immune Inflammatory Indices. He is now exploring the potential ways to mitigate post-stroke Neurological Complications, which seem to share the same immune inflammatory pathomechanisms to PCNS. He thinks it will be fascinating 5-10 years ahead of us. Dr Joanna Laurson-Doube is an International Consultant (Access to Treatment) for the Multiple Sclerosis International Federation (MSIF). She is originally from Finland, but grew up in Belgium and studied and worked in the UK for twenty years before moving to Hong Kong. She has a PhD in Cell Biology from University College London and is currently studying for an Executive MBA at Kellogg-HKUST. She is curious about solving systematic issues in global health and feels passionate about enabling international collaborations for broad impact.

Dr Laurson-Doube, What is your role at MSIF?

[JLD] I work as a consultant for MSIF to improve access to treatment for people with multiple sclerosis (MS), especially in low-resource settings. This could be low and middle-income countries or disadvantaged populations in high income countries, for example uninsured people or refugees. My role at MSIF is to bring together diverse groups of international experts to shape a global strategy to improve access to disease-modifying therapies. We work with our networks (e.g. World Federation of Neurology, European Academy of Neurology, American Academy of Neurology and regional Committees for Treatment and Research in Multiple Sclerosis) and collaborators (e.g. Cochrane MS and rare diseases of the CNS, McMaster University, Medicine Patent Pool) to implement the strategy. I also liaise closely with several teams at the World Health Organisation, e.g. the Brain Health Unit and the Essential Medicine team.

What changes need to be made in MS research and treatment moving forward?

[JLD] MSIF’s Atlas of MS has underlined the serious inequity in accessing MS treatments. Affordability was highlighted as a major barrier by nearly half of the countries surveyed. MS treatments are reported to be too expensive for governments, healthcare or insurance providers, and out-of-pocket costs of disease-modifying therapies for people with MS can lead to catastrophic health expenditure for the family. This is not a simple problem, and I would like to see much more open communication between industry, payers, MS organisations and people with MS to tackle this head-on. It is important that we do not shy away from complexity and confront this on two levels—a pragmatic immediate approach and a sustainable, strategic long-term approach.

Our current project has been pragmatic. We are looking at the ethical use of off-label treatments for MS. ‘Off-label’ treatments have regulatory approval for another indication than MS, for which they are ‘on-label’. A number of off-label treatments for MS are widely used around the world, and they are often more readily available and affordable in health systems. The evidence-base for off-label treatments is different from treatments which have regulatory approval, but off-label treatments can often be the only available option in low-resource settings. The question of whether to use off-label treatments is a real issue that clinicians and people with MS face, and we want to support people to understand the benefits and risks, so they can make the best decision to manage their disease.

In parallel, research into new treatments needs to continue with focused funding efforts, especially for people with MS who have fewer treatment options available. A great example of this is the International Progressive MS Alliance that brings together researchers from across the world and industry to accelerate the development of effective treatments for people with progressive forms of MS.

For any successful campaign that aims to improve global health, raising awareness is essential. This year, World Brain Day is doing just that for MS. Professor Wijeratne, can you please tell us how you got involved with World Brain day and the idea behind it?

[TW] This is an interesting story. I was a Junior Neurologist (second year into my job, preparing myself for a part-time PhD) when I attended a young neurologists and trainees Q&A session. This was at the World Congress Bangkok (WFN) and some of the comments from the speakers regarding the developing world that I had heard were not correct. I grew up in the jungle in Sri Lanka and knew all about hardships, lack of resources etc. I made a few comments from the back row at the end of the workshop and made a few suggestions to address the problem of lack of access and equity in brain health education. That was that and I came home.

A week later, Prof Vladimir Hachinski, the President of WFN then got his PA to send me a letter:

“Dear Dr Wijeratne, the WFN President took note of your comments and he wants you to be the representative member for the Asia Oceania region and action your suggestions to support the region”. I have been heavily involved since then. In 2008, I was browsing the American Academy Neurology website and they were inviting “advocates “ to apply for the Palatucci Advocacy Program. This was like a scholarship with intense training in advocacy, almost like a boot camp (I did not know this- I applied as the criteria they were looking for was basically me). It was a competitive process but I was offered a place. This was game-changing. The boot camp really helped us (30 in the class) to turn our altruistic big ideas into reality. Over the years, the number of advocates they trained grew. Many of us were serving as committee members with WFN. In 2013, Prof Hachinski was telling us about the highly successful World Stroke Brain Day campaign. We were floating with the idea of a World Brain Day. We felt the need and importance of initiatives for brain health. Without brain health we are nothing. A simple chat over a coffee turned into a massive phenomenon the next year. My predecessor, Professor Wasay from Pakistan, was the previous chair of the Global Advocacy committee - I had been an active committee member from the very beginning. The WFN leadership and country delegates from 122 countries unanimously endorsed our idea. The rest is history as you can see from here!

WBD is basically for all people in the world. Brain health is most important. We need to synergise all parties from neurology, psychiatry, rehabilitation, researchers etc…to unify and promote better brain health globally. We now also have a global neurology alliance (GNA), with the same theme and same approach.

What is the reason behind this year’s theme of ‘Stopping MS’?

[TW] It is timely. The pandemic is a strong reminder of neuroinflammation and MS is a great success story. I have interviewed Prof Austin Sumner on this- he worked with Ian McDonald in Dunedin, New Zealand before I was born! The two young men were studying a cat model and how diphtheria toxin was stripping off fat layers around nerve roots—that was the beginning of unravelling underlying mechanisms of disease in the context of this disorder that hits young men and women in their prime. Fast forward 50-60 years and we can almost cure most MS patients now. Yet, access to early diagnosis and better therapy is not possible for 70% of the world. Massive advocacy campaigns are needed to change this. I am very sure we are getting this done.

We are a unique species of living beings and I am eternally optimistic. I think we are cracking most of the mysteries causing havoc in our brain health. Just like the COVID-19 vaccines (thanks to a lot of good brains!), enigmatic disorders such as dementia, Parkinson’s Disease, and Motor Neuron Disease will be cracked during our time. However, we need help! I would love to see one billion people talking about brain health and celebrating world brain day one day. Anyone can visit our website, download the tool box and support and endorse World Brain Day!

Better still—we would love people to do a 15 s video saying “I support the World Brain Day campaign. Let’s stop multiple sclerosis” and you can send your video to wbd2021@wfneurology.org. We are now getting towards spirituality, neurobiology of compassion, love, kindness, meditation etc. Let us survive this pandemic. The most exciting times are ahead of us!

*Interview conducted by Associate Editor Karli Montague-Cardoso for World Brain Day 2021.*

